# Corrosion Resistance Enhancement of the Materials Surface—Second Edition

**DOI:** 10.3390/ma19020383

**Published:** 2026-01-18

**Authors:** Nicanor Cimpoesu, Costică Bejinariu

**Affiliations:** 1 Faculty of Materials Science and Engineering, Gheorghe Asachi Technical University of Iasi, 41 Dimitrie Mangeron Blvd., 700050 Iasi, Romania; nicanor.cimpoesu@academic.tuiasi.ro; 2 Academy of Romanian Scientists, Ilfov 3, 050044 Bucharest, Romania

Understanding and mitigating corrosion remains the main focus regarding extending the service life and reliability of engineering components exposed to aggressive thermal, mechanical and chemical environments. Despite the significant progress made in alloy development, coating systems, inhibitor technologies, and predictive modeling, industrial applications from internal combustion engines to aerospace turbines, marine structures, and chemical processing continue to experience premature failures due to corrosion-assisted degradation [1,2,3]. This Special Issue of *Materials* presents recent advances that combine experimental, computational, and application-driven approaches to improve corrosion resistance, clarify degradation mechanisms, and develop protective materials tailored for modern engineering needs. A key theme emerging from this collection of papers is the shift toward integrated, multiscale approaches to corrosion science. Felix et al. provide an atomistic perspective on stress-corrosion cracking in corundum and hematite using ab initio molecular dynamics, demonstrating that water-assisted dissolution and strain-enhanced detachment processes can occur even at low stress levels, redefining our understanding of subcritical crack growth in protective oxides [4]. Their work fills a critical gap in the mechanistic interpretation of oxide failure, complementing recent studies on dissolution kinetics and crack-tip chemistry [5,6,7]. Such fundamental insights strengthen predictive models for oxide scale degradation in high-temperature alloys [8].

Another significant direction represented in this issue is advanced surface engineering for corrosion protection. Chicet et al. evaluated several atmospheric-plasma-sprayed thermal barrier coatings (TBCs) on engine valves and confirmed that multilayer systems based on Cr_3_C_2_-NiCr, MgZrO_3_-NiCr, and ZrO_2_-CaO markedly improve corrosion resistance both before and after operational exposure [9]. Their findings reinforce ongoing trends in the automotive sector, where TBCs help reduce thermal loads and emissions while increasing component longevity [10]. Complementing this, Gui et al. introduced high-entropy rare-earth zirconates as next-generation TBC materials resistant to calcium–magnesium–alumina–silicate (CMAS) attack, a major degradation pathway in gas turbines [11]. Their demonstration of diffusion-impeding multicomponent chemistries provides a viable route to TBCs capable of sustained operation at temperatures exceeding the stability range of YSZ. Lightweight alloys continue to attract attention due to the ongoing demand for vehicle electrification and energy efficiency. However, their susceptibility to corrosion necessitates innovative protection strategies. Wang et al. proposed a synergistic approach for magnesium alloys, combining sodium tungstate impregnation with silane sealing to enhance micro-arc oxidation (MAO) coatings [12]. Their results show impedance improvements of up to three orders of magnitude, validating tungstate-based inhibitors as environmentally friendly options and echoing developments in smart-release coatings [13]. Similarly, Herrera Hernández et al. demonstrated that Ce–Mo inhibitor treatments significantly improve the pitting corrosion resistance of anodized spray-deposited A390 Al–Si alloy, overcoming the limitations of conventional hot-water sealing and revealing the important role of microstructural refinement on film continuity and inhibitor uptake [14]. The use of rare-earth-based inhibitors in anodic layers and surface oxidation (MAO) demonstrates a principle common to high-entropy ceramic design: exploiting multiple chemical effects to reduce the diffusion of reactive species and stabilize protective phases. In both cases, the simultaneous presence of several elements with different ionic radii leads to the formation of dense layers with reduced atomic mobility and increased resistance to penetration by aggressive environments. This conceptual convergence suggests that entropic strategies can be extended from the field of TBC ceramics to active protection electrochemical systems.

Real-environment degradation rarely arises from corrosion alone; mechanical forces often act synergistically. Mitelea et al. showed that laser remelting of GX40CrNiSi25-20 cast stainless steel refines the surface microstructure, decreases porosity, and substantially improves resistance to both cavitation erosion and electrochemical attack [15]. This work aligns with previous evidence that microstructural homogeneity and carbide dissolution strengthen resistance to mechanically assisted corrosion processes [16,17]. In a different application domain—safety in explosive atmospheres—Chelariu et al. developed a Cu–Al–Be alloy with improved corrosion resistance and reduced tendency to spark during severe frictional loading, offering an alternative to classical Cu–Be tools and expanding the range of materials suited for critical operational environments [18]. Taken together, the contributions in this Special Issue reveal clear development trends and highlight remaining challenges.

Microstructural refinement achieved through LASER resurfacing, MAO layer optimization, or alloy composition control leads, in all cases, to a more homogeneous distribution of phases and a reduction in defects critical for corrosion initiation. The studies analyzed show that fine and compact microstructures simultaneously limit electrochemical processes, mechanical degradation, and crack propagation. This observation highlights a cross-cutting principle of materials design, whereby fine-scale microstructure control is a central tool for improving durability in complex environments.

Future research must continue to integrate atomistic modeling, high-resolution characterization, and long-term environmental testing to better predict corrosion performance over component lifetimes [19,20,21]. There is also a growing need for environmentally benign protection strategies, such as rare-earth-based inhibitors, silane hybrids, and nanocontainer-assisted self-healing systems [22,23]. Furthermore, the rapid advancement of high-entropy ceramics and alloys presents new opportunities for tailoring corrosion and thermal-mechanical responses through controlled chemical complexity [24]. Finally, corrosion studies must increasingly consider synergistic degradation modes—thermal cycling, erosion, cavitation, stress, and chemical exposure—as real service environments rarely involve a single isolated mechanism [25,26]. The atomistic analysis of oxide degradation under the action of water and mechanical stresses provides a fundamental framework for understanding crack initiation and delamination in thermal barrier coating systems, where oxide growth and internal stress accumulation control the lifetime of the protective layer. Studies on conventional TBCs and high-entropy ceramics indicate a clear transition towards multicomponent materials capable of limiting the diffusion of reactive species and chemical degradation at high temperatures. The mechanisms for sealing pores and blocking corrosive environments identified in anodic and MAO layers are conceptually similar to the role of the bond coat in multilayer ceramic systems. The results of laser resurfacing and thermomechanical treatments confirm the importance of a fine and homogeneous microstructure in simultaneously reducing corrosion, cavitation, and wear. Overall, the work highlights the synergistic nature of mechanical and chemical stresses, demonstrating that isolated corrosion assessments are insufficient for predicting behavior under actual operating conditions. These contributions strengthen the link between fundamental mechanisms and applicable engineering solutions, reflecting the maturation of the field of materials protection. The editors thank all contributors for advancing the field and hope that the developments presented here will guide ongoing efforts toward more durable, sustainable, and high-performance material systems.
